# Distortion of double-stranded DNA structure by the binding of the restriction DNA glycosylase R.PabI

**DOI:** 10.1093/nar/gkaa184

**Published:** 2020-03-31

**Authors:** Ken-ichi Miyazono, Delong Wang, Tomoko Ito, Masaru Tanokura

**Affiliations:** Department of Applied Biological Chemistry, Graduate School of Agricultural and Life Sciences, The University of Tokyo, 1-1-1 Yayoi Bunkyo-ku, Tokyo 113–8657, Japan

## Abstract

R.PabI is a restriction DNA glycosylase that recognizes the sequence 5′-GTAC-3′ and hydrolyses the *N*-glycosidic bond of adenine in the recognition sequence. R.PabI drastically bends and unwinds the recognition sequence of double-stranded DNA (dsDNA) and flips the adenine and guanine bases in the recognition sequence into the catalytic and recognition sites on the protein surface. In this study, we determined the crystal structure of the R.PabI-dsDNA complex in which the dsDNA is drastically bent by the binding of R.PabI but the base pairs are not unwound. This structure is predicted to be important for the indirect readout of the recognition sequence by R.PabI. In the complex structure, wedge loops of the R.PabI dimer are inserted into the minor groove of dsDNA to stabilize the deformed dsDNA structure. A base stacking is distorted between the two wedge-inserted regions. R.PabI is predicted to utilize the distorted base stacking for the detection of the recognition sequence.

## INTRODUCTION

**Figure 1.**
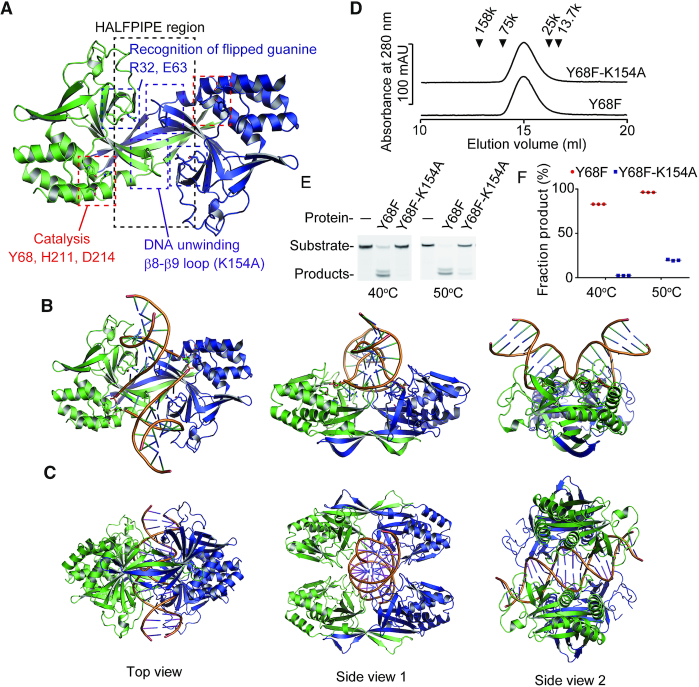
R.PabI–dsDNA complex structures. (**A**) Positions of the important residues/regions of R.PabI. This figure was prepared using the coordinates of R.PabI in complex with product dsDNA (PDB ID: 3WAZ). (**B**) Crystal structure of the R.PabI(K154A)-product dsDNA complex (PDB ID: 3WAZ). The R.PabI protomers are colored green and blue. The dsDNA is coloured orange. The hydrolysed adenine site is shown in stick model. (**C**) Crystal structure of the R.PabI(R32A-E63A)-sequence nonspecific dsDNA complex (PDB ID: 5IFF) (25). (**D**) Gel filtration of the R.PabI(Y68F) (*M*_r_ = 25 344) and the R.PabI(Y68F-K154A) (*M*_r_ = 25 287) mutants. The peak positions of the marker proteins are indicated by black triangles. Both molecular weights of the R.PabI(Y68F) and the R.PabI(Y68F-K154A) mutants were estimated to be 43 000 Da. Data are representative of three independent experiments. The elution volume of each experiment was approximately the same. (**E**) DNA glycosylase activities of the R.PabI(Y68F) and the R.PabI(Y68F-K154A) mutants at 40°C and 50°C. Data are representative of three independent experiments. (**F**) Quantification of (E). The fractions of products from the three independent experiments are indicated as red circles (R.PabI(Y68F)) and blue squares (R.PabI(Y68F-K154A)). The mean values are shown by black lines. The fractions of products at 40°C are 82.93 ± 0.03% (R.PabI(Y68F)) and 2.38 ± 0.05% (R.PabI(Y68F-K154A)) (the mean ± SEM). The fractions of products at 50°C are 96.33 ± 0.08% (R.PabI(Y68F)) and 19.78 ± 0.31% (R.PabI(Y68F-K154A)) (the mean ± SEM).

Restriction-modification systems, which consist of restriction enzymes and DNA methyltransferases, play important roles in protecting genomes from foreign DNA. Restriction enzymes recognize specific DNA sequences that are not modified by their cognate DNA methyltransferases and introduce double-strand breaks into DNA. Because most restriction enzymes hydrolyse phosphodiester bonds to cleave DNA, they are also called restriction endonucleases (1). Among restriction enzymes, type II restriction enzymes recognize specific double-stranded DNA (dsDNA) sequences and cleave dsDNA at or near the recognition sequences. Type II restriction enzymes are structurally classified into five groups (2): the PD-(D/E)XK superfamily (3–5), the HNH superfamily (6,7), the PLD superfamily (8), the GIY-YIG superfamily (9) and the HALFPIPE superfamily (10). Among these superfamilies, the PD-(D/E)XK superfamily, the HNH superfamily and the GIY-YIG superfamily proteins require Mg^2+^ ions to hydrolyse phosphodiester bonds. In contrast, the PLD superfamily and the HALFPIPE superfamily proteins cleave dsDNA in Mg^2+^ ion-independent manners. The PLD superfamily proteins cleave dsDNA using a phospholipase D-like active site that hydrolyses the phosphodiester bond of dsDNA in a Mg^2+^ ion-independent manner (8). On the other hand, our previous study revealed that the HALFPIPE superfamily proteins are not endonucleases but DNA glycosylases that hydrolyse *N*-glycosidic bonds that link the base and deoxyribose moieties of DNA (11). R.PabI from the hyperthermophilic archaeon *Pyrococcus abyssi* was the first HALFPIPE superfamily enzyme discovered (12). R.PabI recognizes the sequence 5′-GTAC-3′ of dsDNA at its electropositive HALFPIPE region and flips adenine and guanine bases in the recognition sequence to outside of the DNA helix using its β8-β9 loop. R.PabI hydrolyses the *N*-glycosidic bond of the flipped adenine using three catalytic residues (Y68, H211 and D214) (Figure 1A), Y68 is used to stabilize the catalytic water; H211 is used to localize the position of Y68; and D214 is used to stabilize the oxocarbenium ion intermediate and to deprotonate the catalytic water. Two opposing apurinic/apyrimidinic (AP) sites are cleaved by β-elimination and/or other AP endonucleases (11).

Structures of dsDNA are frequently modified by interactions of DNA-binding proteins. For example, the TATA-binding protein, which plays a critical role in transcription, binds to the minor groove region of dsDNA in the TATA box sequence using its β sheet structure and bends dsDNA by ∼80° (13). The structures of dsDNA are also modified by the binding of various DNA binding proteins, such as transcription factors (14,15), restriction endonucleases (16–19), DNA methyltransferases (20) and DNA repair enzymes, such as endonucleases and DNA glycosylases (21–24). Distortions of dsDNA structures are indispensable for these enzymes to recognize specific sequences and/or catalyse specific reactions at specific sites in dsDNA. For example, the restriction endonuclease ThaI distorts the structure of dsDNA at its recognition sequence by inserting amino acid residues into base stacks (17). The 8-oxoguanine glycosylase hOGG1 flips a DNA base out of the dsDNA helix to recognize and cleave a damaged base (22). Similarly to these enzymes, R.PabI also drastically modifies the structure of dsDNA for the recognition and cleavage of specific site of dsDNA. The crystal structure of the R.PabI–dsDNA complex showed that R.PabI bends dsDNA by ∼90° and unwinds dsDNA at the recognition sequence (5′-GTAC-3′) (Figure 1B) (11). The crystal structure of the R.PabI-sequence nonspecific-dsDNA complex showed that R.PabI forms a tetrameric structure on dsDNA to efficiently find the recognition sequence (Figure 1C) (25). In contrast to the drastic structural change of dsDNA observed in the R.PabI–product–dsDNA complex, the dsDNA structure in the R.PabI-sequence nonspecific-dsDNA complex is similar to typical B-form dsDNA, although the minor groove width of the dsDNA is slightly widened by the binding of R.PabI. The mechanism by which R.PabI distorts dsDNA from a B-form-like structure to the characteristic bent and unwound dsDNA structure remains unclear. The structure determination of the intermediate state between the product dsDNA-binding state and the sequence-nonspecific dsDNA-binding state is required to clarify the DNA bending and unwinding mechanism by R.PabI.

Here, we report the crystal structures of the R.PabI(Y68F-K154A) mutant in complex with three types of dsDNA (Supplementary Figure S1). The complex structures show that the dsDNA structures in the complexes are drastically bent by the binding of R.PabI similar to the structure of the R.PabI–product–dsDNA complex (11), although the base-pairs are not unwound. The β2-β3 loop of R.PabI is inserted into the minor groove of dsDNA like a ‘wedge’ to stabilize the largely expanded minor groove. The base stacking of dsDNA is distorted at the expanded minor groove region in a position-dependent manner. R.PabI is predicted to unwind the dsDNA in the recognition sequence using this distortion of base stacking.

## MATERIALS AND METHODS

### Protein expression and purification

The gene fragment of R.PabI (residues 8–226) was cloned into the pET26b vector (Novagen). The expression vectors of various R.PabI mutants (R.PabI(Y68F), R.PabI(Y68F-K154A), R.PabI(Y68F-P27G), R.PabI(Y68F-P27G-T28G), R.PabI(K154A) and R.PabI(K154A-P27G-T28G)) were prepared using the PrimeSTAR Mutagenesis Basal Kit (TaKaRa). For protein expression, the constructed plasmids were transformed into *Escherichia coli* Rosetta(DE3)pLysS cells (Novagen). The recombinant *E. coli* cells were cultivated in LB medium supplemented with kanamycin (20 μg/ml) and chloramphenicol (50 μg/ml) at 37°C until the optical density of the medium at 600 nm reached 0.6. Protein expression was induced by the addition of 1 mM isopropyl-β-d-thiogalactopyranoside (IPTG). After overnight cultivation at 25°C, cells were harvested by centrifugation.

*Escherichia coli* cells overexpressing the R.PabI mutants were resuspended in 25 mM MES pH 6.0 and 50 mM MgCl_2_, and the cells were disrupted by sonication. After centrifugation at 40 000 × g for 30 min at 4°C, the supernatant was treated with Cryonase Cold-Active Nuclease (TaKaRa) to remove contaminant nucleic acids from *E. coli*. The solution was heated at 80°C for 30 min to denature the heat-unstable *E. coli* proteins. After centrifugation at 40 000 × g for 30 min at 4°C, the supernatant was purified by TOYOPEARL AF-Heparin HC-650 (TOSOH) resin and a Mono S 10/10 (GE Healthcare) column. Purified proteins were stored at −80°C until use.

### Crystallization and structure determination

The R.PabI(Y68F-K154A) mutant was mixed with three types of dsDNA, dsDNA(GTAC-3 bp-GTAC), dsDNA(GTAC-5 bp-GTAC) and dsDNA(nonspecific) (Supplementary Figure S1A), in 10 mM MES pH 6.0 and 100 mM NaCl (the molar ratio of the R.PabI dimer and dsDNA was 1:1) and concentrated to 180 μM (the concentration of the R.PabI dimer). Crystallization of the R.PabI-dsDNA complexes was performed using the sitting-drop vapor-diffusion method at 20°C. The crystal of the R.PabI(Y68F-K154A)-dsDNA(GTAC-3 bp-GTAC) complex was obtained under reservoir solution conditions containing 0.1 M Bis–Tris pH 5.9 and 51% 2-methyl-2,4-pentanediol (MPD). The crystal of the R.PabI(Y68F-K154A)-dsDNA(GTAC-5 bp-GTAC) complex was obtained under reservoir solution conditions containing 0.1 M Bis–Tris pH 6.3 and 51% MPD. The crystal of the R.PabI(Y68F-K154A)-dsDNA(nonspecific) complex was obtained under reservoir solution conditions containing 0.1 M Bis–Tris pH 6.0 and 50% MPD. X-ray diffraction data of the dsDNA(GTAC-3 bp-GTAC) and dsDNA(GTAC-5 bp-GTAC) complexes were collected on the BL-17A beamline at the Photon Factory (Tsukuba, Japan) at 95 K. X-ray diffraction data of the dsDNA(nonspecific) complex were collected on the AR-NW12A beamline at the Photon Factory (Tsukuba, Japan) at 95 K. The crystal of the R.PabI(Y68F-K154A)-dsDNA complexes diffracted X-rays to 2.45 Å (the dsDNA(GTAC-3 bp-GTAC) complex), 2.20 Å (the dsDNA(GTAC-5 bp-GTAC) complex) and 2.75 Å (the dsDNA(nonspecific) complex). The X-ray diffraction data were indexed, integrated and scaled with XDS (26). The crystal of the dsDNA(GTAC-3 bp-GTAC) complex belongs to the space group *P*3_2_21 with unit cell parameters of *a* = 83.68 Å, *b* = 83.68 Å and *c* = 140.35 Å. The crystal of the dsDNA(GTAC-5 bp-GTAC) complex belongs to the space group *P*3_2_21 with unit cell parameters of *a* = 82.43 Å, *b* = 82.43 Å and *c* = 140.35 Å. The crystal of the dsDNA(nonspecific) complex belongs to the space group *P*3_2_21 with unit cell parameters of *a* = 84.15 Å, *b* = 84.15 Å and *c* = 140.53 Å. The initial models of the complexes were determined with MOLREP (27) in the CCP4 suite (28) using the coordinates of R.PabI (PDB ID: 5IFF) (25) as a search model. The structures of the complexes were refined and rebuilt using the programs Phenix.refine (29) and Coot (30). The geometry of the final model was evaluated with MolProbity (31). The data collection and refinement statistics are summarized in Table 1.

**Table 1.** Summary of data collection and refinement statistics of the R.PabI(Y68F-K154A)-dsDNA complexes

	dsDNA (GTAC-3 bp-GTAC)	dsDNA (GTAC-5 bp-GTAC)	dsDNA (nonspecific)
**Data collection**			
Space group	*P*3_2_21	*P*3_2_21	*P*3_2_21
Cell dimensions			
*a*, *b*, *c* (Å)	83.68, 83.68, 140.35	82.43, 82.43, 140.35	84.15, 84.15, 140.53
Resolution (Å)	41.84–2.45 (2.55–2.45)	46.78–2.20 (2.27–2.20)	46.84–2.75 (2.90–2.75)
*R*_sym_ (%)	4.2 (65.3)	6.8 (78.5)	5.3 (61.4)
Mean (*I*/σ*I*)	16.0 (1.9)	14.4 (1.7)	11.8 (1.4)
Completeness (%)	97.7 (86.8)	100 (99.9)	99.0 (99.0)
Redundancy	4.7 (4.5)	10.1 (6.7)	4.3 (4.3)
CC (1/2)	0.999 (0.866)	0.999 (0.832)	0.999 (0.857)
**Refinement**			
*R*/*R*_free_ (%)	22.88/25.57	21.66/25.72	28.33/30.65
No. atoms			
R.PabI-chain A	1766	1766	1735
R.PabI-chain B	1717	1728	1361
DNA	469	407	246
*B*-factors (Å^2^)			
R.PabI-chain A	73.02	73.15	102.06
R.PabI-chain B	92.66	85.38	130.62
DNA	100.54	109.03	170.98
R.m.s deviations			
Bond lengths (Å)	0.003	0.004	0.004
Bond angles (°)	0.602	0.623	0.658
Ramachandran plot			
Favoured region (%)	97.2	96.9	96.2
Allowed region (%)	99.8	100	100

The numbers in parentheses represent data for the highest-resolution shells.

### Gel filtration analysis

The R.PabI mutants (2 μM as a dimer) were loaded onto a Superdex 200 HR 10/30 (GE Healthcare) column and eluted with buffer containing 10 mM MES pH 6.0 and 500 mM NaCl. To estimate the oligomeric state of the R.PabI mutants, the following standard proteins were used: aldolase (*M*_r_ = 158 000), conalbumin (*Mr* = 75 000), chymotrypsinogen A (*M*_r_ = 25 000) and ribonuclease A (*M*_r_ = 13 700).

### Enzymatic assay

DNA glycosylase activity assays of the R.PabI mutants were performed using 24 bp dsDNA possessing one R.PabI recognition sequence at the centre. A 5′-fluorescein-labeled 24 bp dsDNA (5′-fluorescein-GGATGCATGAGTACGAGGACCATC-3′, Supplementary Figure S1B) was purchased from Eurofins Genomics. Substrate dsDNA (0.2 μM) was mixed with 0.4 μM R.PabI dimers in a reaction buffer containing 0.1 M phosphate buffer pH 6.5. The reaction solutions were incubated at 40°C and 50°C for 120 min or at 40°C for 5, 10, 15, 20, 30, 60, 90 and 120 min. After the enzymatic reactions, the reaction solutions were supplemented with 0.1 M NaOH to stop the enzymatic reaction. The solutions were then heated at 70°C for 10 min to cleave the products at the 5′ and 3′ sides of the AP sites generated by R.PabI and neutralized by the addition of an equal concentration of HCl. The reaction solutions were separated on a denaturing 18% polyacrylamide gel in 0.5× TBE and 7 M urea. The fluorescence was measured using an Amersham Imager 680 (GE Healthcare) and was quantified with Amersham Imager 680 Analysis Software (GE Healthcare). The enzymatic rate constant *k*_obs_ was obtained from a single-exponential fit to the data from three independent measurements: *f*_p_ = *f*_p_max × (1 − e^−^*^kt^*), where *f*_p_ is the fraction of product, *f*_p__max_ is the maximum value of *f*_p_, and *t* is the time of the reaction (min).

### Electrophoretic mobility shift assay (EMSA)

EMSA experiments were performed as described previously with minor modifications (25). The 5′-fluorescein-labeled 24 bp dsDNA (5′-GGATGCATGAGTACGAGGACCATC-3′ (the specific probe) and 5′-GGATGCATGAGATCGAGGACCATC-3′ (the nonspecific probe)) were used as probes (Supplementary Figure S1BC). Then, 0.1 μM of the DNA probe and the R.PabI (Y68F and its mutants) dimer were mixed in 10 mM MES pH 6.0 and 300 mM NaCl in the presence of a 25-fold excess amount of an unlabelled competitor dsDNA (5′-GGATGCATGAGATCGAGGACCATC-3′). In addition, 0.1 μM of the DNA probe and the R.PabI (K154A and its mutants) dimer were mixed in the same buffer in the presence of a 50-fold excess amount of the unlabeled competitor dsDNA. The samples were separated using a 10% polyacrylamide gel in 0.5 × TBE at 4°C. The fluorescence was measured using an Amersham Imager 680 (GE Healthcare) and was quantified with Amersham Imager 680 Analysis Software (GE Healthcare).

### Computational analysis

The structures of the R.PabI-dsDNA complexes were analysed using the following set of computer programs: Clustal Omega for the amino acid sequence alignment (32); ESpript for the preparation of alignment figures (33); APBS for the calculation of macromolecular electrostatics (34); Curves+ for the analysis of dsDNA structure (35) and PyMOL (https://www.pymol.org/) for the depiction of the structures.

## RESULTS

### Overall structure of the R.PabI–dsDNA complexes

**Figure 2.**
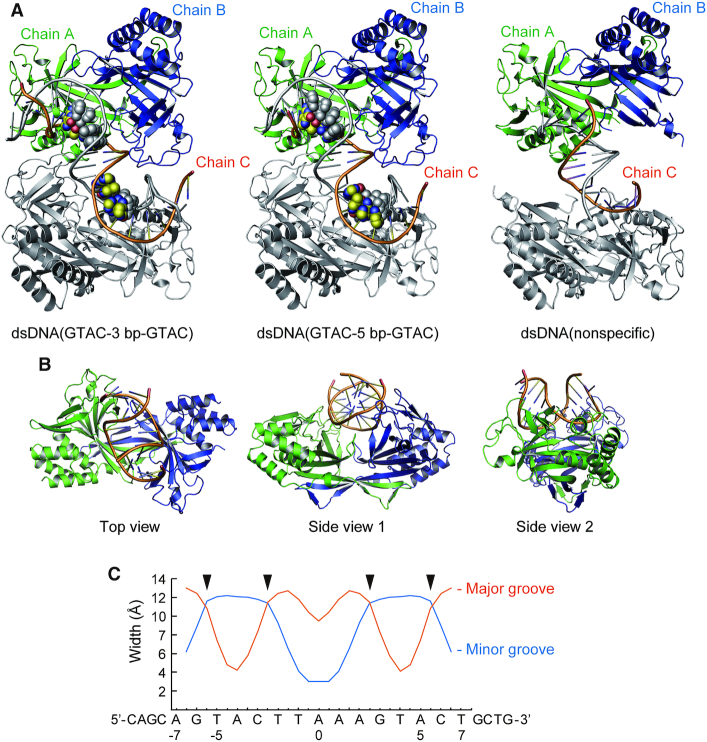
Overall structures of the R.PabI(Y68F-K154A)–dsDNA complexes. (**A**) Structures of the R.PabI(Y68F-K154A)–dsDNA(GTAC-3 bp-GTAC), the R.PabI(Y68F-K154A)–dsDNA(GTAC-5 bp-GTAC) and the R.PabI(Y68F-K154A)–dsDNA(nonspecific) complexes in crystals. The R.PabI protomers in the asymmetric unit (chains A and B) are colored green and blue, respectively. The bound DNA strand (chain C) is coloured orange. The R.PabI–dsDNA complex structures generated by a crystallographic 2-fold axis are colored grey. The 5′-GTAC-3′ sequence in the dsDNA is shown in sphere models. (**B**) Top and side views of the R.PabI(Y68F-K154A)–dsDNA(GTAC-5 bp-GTAC) complex. (**C**) Major and minor groove widths of dsDNA in the R.PabI(Y68F-K154A)–dsDNA(GTAC-5 bp-GTAC) complex. The positions at which the wedge loops (β2–β3) are inserted are marked with black triangles.

In this study, we utilized the Y68F-K154A double mutant of R.PabI to determine the structures of the R.PabI-dsDNA complex. K154A of R.PabI is located in the β8–β9 loop, which is utilized for base flipping by R.PabI (Figure 1A). The K154A mutant of R.PabI, which exhibits reduced sequence-specific DNA binding and DNA cleavage activities, has been used to determine the crystal structure of the R.PabI–product–dsDNA complex (11). Y68 is a catalytic residue of R.PabI and the Y68F mutant has approximately 1% activity compared to the wild-type enzyme, although the mutant retains approximately the same sequence-specific DNA binding ability as the wild-type R.PabI (11). The Y68F-K154A double mutant of R.PabI formed a homodimer similar to wild-type R.PabI and showed no cleavage activity at 40°C, although the Y68F-K154A double mutant showed weak activity at 50°C (Figure 1D–F). These results show that the R.PabI(Y68F-K154A) mutant possesses DNA glycosylase activity, but the activity is highly reduced. In the course of the cocrystallization experiments of R.PabI(Y68F-K154A) and dsDNA, we obtained low-quality crystals of R.PabI(Y68F-K154A)–dsDNA complexes when we used a 23 bp dsDNA that did not contain the 5′-GT-3′, 5′-TA-3′ or 5′-AC-3′ steps (dsDNA(nonspecific), Supplementary Figure S1A). Because the R.PabI(Y68F-K154A)-dsDNA(nonspecific) complex structure showed that two R.PabI dimers bind to one dsDNA, we designed the dsDNA sequences that possess the R.PabI recognition sequence (5-GTAC-3′) near each R.PabI binding site (dsDNA(GTAC-3 bp-GTAC) and dsDNA(GTAC-5 bp-GTAC), Supplementary Figure S1A). When we used these dsDNA fragments, we obtained high-quality crystals of R.PabI(Y68F-K154A)–dsDNA complexes. In this study, we determined the structures of R.PabI(Y68F-K154A) in complex with dsDNA(GTAC-3 bp-GTAC), dsDNA(GTAC-5 bp-GTAC) and dsDNA(nonspecific) at 2.45, 2.20 and 2.75 Å resolutions, respectively. Each complex contains one R.PabI dimer (chains A and B) and one DNA strand (chain C) in the asymmetric unit (Supplementary Figure S2). In the R.PabI(Y68F-K154A)–dsDNA(GTAC-3 bp-GTAC) complex structure, structure models of amino acid residues 224–226 of chains A and B and those of amino acid residues 13–17 of chain B are not included in the final model due to low electron density. In the R.PabI(Y68F-K154A)–dsDNA(GTAC-5 bp-GTAC) complex, structure models of amino acid residues 224–226 of chains A and B, amino acid residues 12–16 of chain B, and DNA residues −11, −10 and 11 are not included in the final model. In the R.PabI(Y68F-K154A)–dsDNA(nonspecific) complex, structure models of amino acid residues 156–158 and 224–226 of chain A, amino acid residues 8–26, 41–53, 173–190 and 223–226 of chain B, and DNA residues −11 to −8 and 5 to 11 are not included in the final model. Each DNA strand in the R.PabI(Y68F-K154A)–dsDNA complexes interacts with a symmetrically related DNA strand to form dsDNA; in crystal, two R.PabI dimers interact with one dsDNA (Figure 2A and Supplementary Figure S3A). There is no interprotein contact between the two R.PabI dimers that bind the same dsDNA, indicating that the R.PabI structures observed in this study are dimers, not a tetramer. Although the DNA sequences used for cocrystallization are not identical, the structures of the R.PabI(Y68F-K154A)-dsDNA complexes are nearly identical; the maximal root mean square deviation (RMSD) among the complexes was 0.304 Å for 395 superposed Cα atoms in the asymmetric unit (between the dsDNA(GTAC-3 bp-GTAC) complex and the dsDNA(GTAC-5 bp-GTAC) complex). In addition, the backbone structures of dsDNA are approximately the same among the complexes (Supplementary Figure S2A). These structural similarities among the complexes indicate that the structures observed in this study are rarely affected by dsDNA sequences. Due to the poor electron density, the DNA structure in the R.PabI(Y68F-K154A)-dsDNA(nonspecific) complex was not precisely determined. Therefore, we used the structures of the R.PabI(Y68F-K154A)–dsDNA(GTAC-3 bp-GTAC) and R.PabI(Y68F-K154A)–dsDNA(GTAC-5 bp-GTAC) complexes to analyse the R.PabI–DNA interactions. Because the structure of the R.PabI(Y68F-K154A)–dsDNA(GTAC-5 bp-GTAC) complex was determined at higher resolution, we used the structure of the R.PabI(Y68F-K154A)–dsDNA(GTAC-5 bp-GTAC) complex for the figures unless otherwise stated.

In the HALFPIPE region, the minor groove side of dsDNA is largely expanded by the binding of R.PabI (Figure 2B); the minor groove width of this region is ∼12 Å, although that of typical B-form dsDNA is 5.9 Å. Accordingly, the major groove width of the corresponding region is reduced to 4 Å; that of typical B-form dsDNA is 11.4 Å (Figure 2C). The dsDNA structures in the R.PabI(Y68F-K154)–dsDNA complexes are distinct from those in the sequence-nonspecific dsDNA-binding state and the product dsDNA-binding state (11,25) (Figures 1B, C and 2B). Although the dsDNA structure is drastically bent by the binding of R.PabI(Y68F-K154A), similar to that in the R.PabI-product dsDNA complex (11), the dsDNA structure in the R.PabI(Y68F-K154A)–dsDNA complex is not unwound, similar to that in the sequence-nonspecific dsDNA binding state (25). These observations suggest that the dsDNA structures observed in the R.PabI(Y68F-K154A)–dsDNA complexes represent the intermediate state between the sequence-nonspecific dsDNA-binding state and the product dsDNA-binding state. Hereafter, we designate the dsDNA structures observed in this study as the intermediate state.

### Structure modification of R.PabI

**Figure 3.**
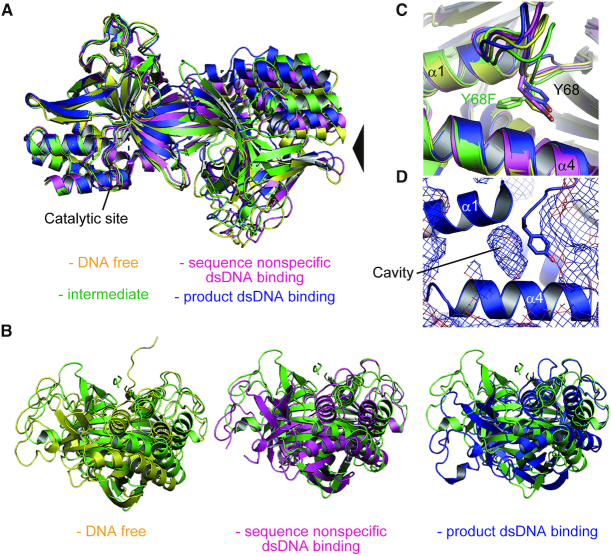
Structural comparison of the R.PabI dimers. (**A**) Superposition of the R.PabI structures in the DNA-free state (PDB ID: 2DVY, yellow), the sequence-nonspecific dsDNA-binding state (PDB ID: 5IFF, magenta), the intermediate state (green) and the product dsDNA-binding state (PDB ID: 3WAZ, blue). The structures were superposed using the coordinates of chain A structures. The positions of the catalytic sites are indicated by black dotted squares. (**B**) Side views of (A). The viewpoint is indicated by a black triangle in (A). (**C**) Side chain flipping of Y68F observed in the intermediate state. The side chains of Y68 and Y68F are shown by stick models. (**D**) Cavity adjacent to Y68. The protein surface of R.PabI (PDB ID: 3WAZ) is shown in mesh.

To analyze the structural modification of R.PabI by binding dsDNA, the structures of R.PabI dimers in the DNA-free state (PDB ID: 2DVY) (10), the sequence-nonspecific dsDNA-binding state (PDB ID: 5IFF) (25), the product dsDNA-binding state (PDB ID: 3WAZ) (11) and the intermediate state were superposed using the coordinates of their R.PabI protomers (Figure 3A, B and Supplementary Figure S3B). The protomer structures of R.PabI in each state are nearly identical; the maximal RMSD between protomers is 0.843 Å for 171 superposed Cα atoms (between the DNA-free state and the product dsDNA-binding state). In contrast, the dimeric structures in each state are modified by the binding of dsDNA (Figure 3A, B). The dimerization of R.PabI is mainly stabilized by the β-sheet formation in the HALFPIPE region, R.PabI protomers in R.PabI dimers are easy to twist in the interfacing region. The structural comparison of the R.PabI dimers showed that the R.PabI protomer is most twisted in a clockwise direction at the intermediate state (Figure 3B).

In this study, we utilized the Y68F-K154A double mutant of R.PabI to determine the dsDNA complex structure. Y68 is the catalytic residue of R.PabI (Figure 1A). The structure of the R.PabI(Y68F-K154A)-dsDNA complexes shows that the side chain of Y68F flips into the inner side of the protein (Figure 3C). In the R.PabI-product-dsDNA complex structure, there is a cavity adjacent to the side chain of Y68 (Figure 3D); the side chain of Y68F flips into this cavity. The side chain flipping of Y68F is predicted to occur due to the lack of the side chain hydroxyl group of Y68.

### R.PabI–dsDNA interaction

**Figure 4.**
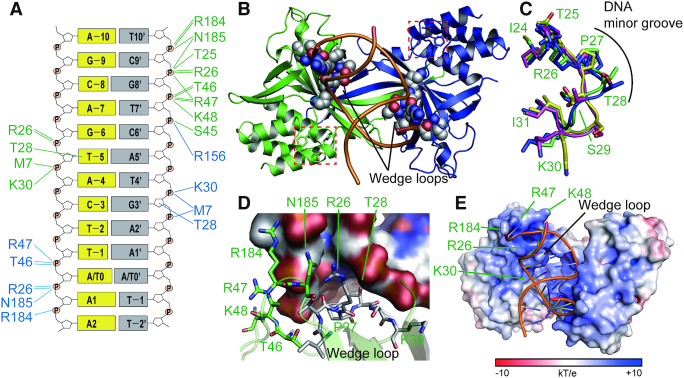
dsDNA recognition by the R.PabI(Y68F-K154A) mutant. (**A**) Intermolecular hydrogen bonds between R.PabI(Y68F-K154A) and dsDNA. Residues of chains A and B are shown in green and blue texts, respectively. Intermolecular hydrogen bonds between R.PabI and DNA are shown in green (R.PabI chain A-dsDNA) and blue (R.PabI chain B-dsDNA) lines. (**B**) Insertion of the wedge loop (β2-β3) of R.PabI into the minor groove of dsDNA. Residues in the wedge loop are shown in sphere models. The catalytic residues (Y68F, H211 and D214) are shown by stick models and are indicated by red dotted boxes. (**C**) Structure comparison of the wedge loop. Residues of the DNA-free state (PDB ID: 2DVY), the sequence-nonspecific dsDNA-binding state (PDB ID: 5IFF), the intermediate state and the product dsDNA-binding state (PDB ID: 3WAZ) are colored yellow, magenta, green and blue, respectively. The position of the DNA minor groove is indicated by a black line. (**D**) R.PabI residues adjacent to the largely expanded minor groove. Residues in the wedge loop are shown in grey stick models. Other R.PabI residues are shown in green stick models. (**E**) DNA backbone binding by the positively charged surface of R.PabI. The ±10 kT/e electrostatic potential of R.PabI is plotted on the surface.

The R.PabI(Y68F-K154A) homodimers mainly interact with phosphate groups of dsDNA (Figure 4A, Supplementary Figure S4 and Supplementary Tables S1 and S2). The DNA backbone recognition mechanisms observed in the structures of the R.PabI(Y68F-K154A)–dsDNA(GTAC-5 bp-GTAC) and the R.PabI(Y68F-K154A)–dsDNA(GTAC-3 bp-GTAC) complexes are nearly identical, except for the residues around the β8–β9 loop. The complex structures show that the β8-β9 loops possess higher temperature factors than the other parts of R.PabI, indicating that the β8–β9 loops are relatively flexible (Supplementary Figure S2C). Therefore, although the β8-β9 loop is indispensable for the base recognition by R.PabI (11), it remains unclear whether or not the differences in the R.PabI–DNA backbone interactions around the β8-β9 loop are important for the stabilization of the intermediate state. In the R.PabI(Y68F-K154A)–dsDNA(GTAC-5 bp-GTAC) complex and R.PabI(Y68F-K154A)–dsDNA(GTAC-3 bp-GTAC) complex structures, R.PabI dimers form 31 and 32 hydrogen bonds with the bound dsDNA structures, respectively. By these R.PabI-DNA interactions, the structure of dsDNA is drastically bent compared to typical B-form DNA (Figure 2B, C). To stabilize the bent dsDNA structure, R.PabI drives the β2-β3 loop like a ‘wedge’ into the minor groove of dsDNA (Figure 4B). The minor groove width of dsDNA is largely expanded between the positions at which the two wedges of the R.PabI homodimers are driven (Figure 2C). Hereafter, we designate the β2–β3 loop as the wedge loop. In the R.PabI(Y68F-K154A)–dsDNA complex structures, the side chain atoms of P27 and T28 of the wedge loop are inserted deeply into the minor groove of dsDNA (Figure 4C, D and Supplementary Figure S3C). Because the side chain of T28 is inserted into the minor groove of dsDNA, the side chain hydroxyl group of T28 forms direct hydrogen bonds with the base and sugar groups of Thy−5 and Gua3′ in the dsDNA(GTAC-5 bp-GTAC) complex and with base groups of Gua-5 and Gua2′ in the dsDNA(GTAC-3 bp-GTAC) complex (DNA bases of the symmetrically related molecules are indicated by a prime (Figure 4A)). The structure of the wedge loop is modified to stabilize the bent dsDNA structure. In contrast to the structures in the DNA-free and the sequence-nonspecific dsDNA-binding states, the side chain of T28 is protruded into the bound dsDNA in the intermediate state; the wedge loop structure is maintained in the product dsDNA-binding state (Figure 4C). These observations suggest that the wedge loop structures that are observed in the intermediate and the product dsDNA-binding states are utilized for stabilization of the largely expanded minor groove structure.

Around the wedge loop region, R.PabI possesses positively charged residues such as R26, K30, R47, K48 and R184 to bind the negatively charged DNA backbone (Figure 4D). Among these residues, R47 and R184 are also utilized for binding the DNA backbone in the product dsDNA-binding state (11): K30, R47, K48 and R184 are also utilized for binding the DNA backbone in the sequence-nonspecific dsDNA-binding state (25) (Supplementary Figure S5). These observations suggest that R.PabI can bind the various structures of dsDNA (Figures 1A, B and 2B) in the HALFPIPE region. The electrostatic potential of the R.PabI(Y68F-K154A) surface shows that the wedge loop region of R.PabI possesses a positively charged surface (Figure 4E). A negatively charged dsDNA backbone is predicted to slide on the positively charged R.PabI surface to change the bound dsDNA structure.

### Distortion of base stacking

**Figure 5.**
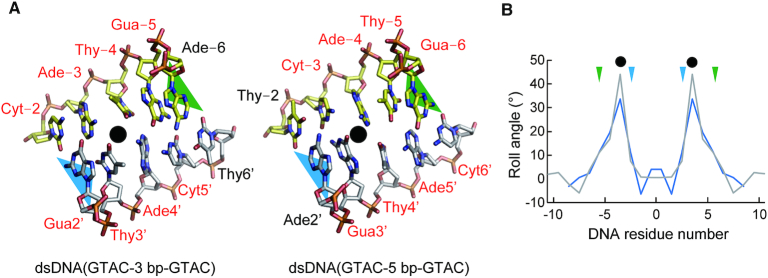
Distortion of the base stacking. (**A**) Distorted base stackings in the R.PabI(Y68F-K154A)-dsDNA complexes. DNA bases are shown in stick models. The positions of the wedge loops are shown by green (chain A) and blue (chain B) triangles. The positions of distorted base stackings are indicated by black circles. The DNA residues of the 5′-GTAC-3′ sites are labelled by red font. (**B**) Roll angles of dsDNA in the dsDNA(GTAC-3 bp-GTAC) complex (gray) and the dsDNA(GTAC-5 bp-GTAC) complex (blue). The wedge loop-inserted regions are marked with triangles. The positions of distorted base stackings are indicated by black circles.

Generally, dsDNA structures are stabilized by base stacking interactions between adjacent bases. In contrast, the base stackings in the largely expanded minor grove regions in the R.PabI(Y68F-K154A)–dsDNA complexes are distorted (Figure 5 and Supplementary Figure S2B). Because the DNA sequences of the expanded minor groove regions are not identical between dsDNA(GTAC-3 bp-GTAC) and dsDNA(GTAC-5 bp-GTAC) (Supplementary Figure S1A), the distortions of base stacking are predicted to occur in a sequence-independent manner and in a position-dependent manner. In the R.PabI(Y68F-K154A)–dsDNA complex structures, the wedge loops are inserted between DNA bases −5 and −6 and between DNA bases 2′ and 3′ (Figure 5); there are three base pairs and two base stackings between the two wedge loops. In each complex, the roll angles between DNA bases −3 and −4 are largely increased (Figure 5B). In addition, the roll angles between DNA bases −4 and −5 are also increased. Because the DNA bases of these regions do not form direct interactions with R.PabI, the distortion of base stacking is predicted to be induced by the DNA bending and the minor groove expansion that are stabilized by the wedge loop insertions.

### Mutation assay

**Figure 6.**
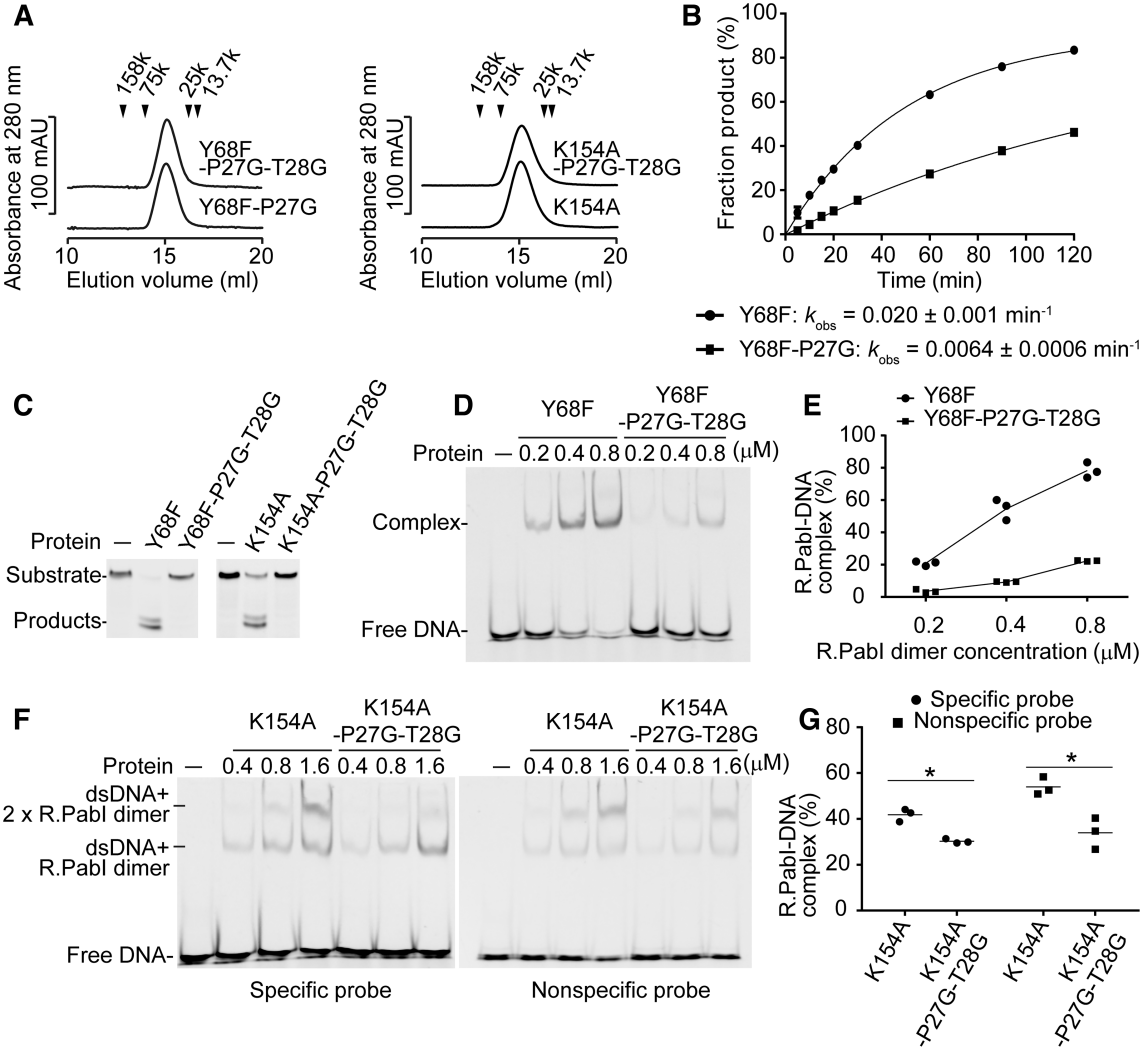
Mutation assays. (**A**) Gel filtration of the R.PabI(Y68F-P27G) (*M*_r_ = 25 304), the R.PabI(Y68F-P27G-T28G) (*M*_r_ = 25 260), the R.PabI(K154A) (*M*_r_ = 25 303) and the R.PabI(K154A-P27G-T28G) (*M*_r_ = 25 219) mutants. The peak positions of the marker proteins are indicated by black triangles. Molecular weights of R.PabI(Y68F-P27G), R.PabI(Y68F-P27G-T28G), R.PabI(K154A) and R.PabI(K154A-P27G-T28G) were estimated to be 41 000, 41 000, 44 000 and 43 000 Da, respectively. Data are representative of three independent experiments. The elution volume of each experiment was approximately the same. (**B**) Kinetics experiments for the DNA glycosylase activities of the R.PabI(Y68F) and the R.PabI(Y68F-P27G) mutants at 40°C. The enzymatic rate constant and its standard error were obtained from a single-exponential fit to the data from three independent measurements. Plotted values are the mean ± SEM (*n* = 3). (**C**) DNA glycosylase activities of the R.PabI(Y68F), the R.PabI(Y68F-P27G-T27G), the R.PabI(K154A) and the R.PabI(K154A-P27G-T28G) mutants at 50°C. Data are representative of three independent experiments. (**D**) EMSA of the R.PabI(Y68F) and the R.PabI(Y68F-P27G-T28G) mutants using the specific probe (Supplementary Figure S1B). 100 nM of the fluorescein-labelled dsDNA and each concentration of the R.PabI dimer were mixed and separated using a 10% polyacrylamide gel. Data are representative of three independent experiments. (**E**) Quantification of (D). The fractions of R.PabI–DNA complexes are indicated by circles (R.PabI(Y68F)) and squares (R.PabI(Y68F-P27G-T28G)). Lines connect the mean value of each data. (**F**) EMSA of the R.PabI(K154A) and R.PabI(K154A-P27G-T28G) mutants using the specific probe and the nonspecific probe (Supplementary Figure S1C). 100 nM of the fluorescein-labeled dsDNA and each concentration of the R.PabI dimer were mixed and separated using a 10% polyacrylamide gel. Data are representative of three independent experiments. (**G**) Quantification of (F). The fractions of complexes (at 1.6 μM of the R.PabI dimer) are indicated by circles (the specific probe) and squares (the nonspecific probe). The mean values are shown by black lines. *P < 0.05; Student's *t*-test.

The R.PabI(Y68F-K154A)–dsDNA complex structures show that the wedge loops of R.PabI dimers are important to maintain the distorted dsDNA structure. To analyse the importance of the structural rigidity of the wedge loops, we prepared the Y68F-P27G double mutant and the Y68F-P27G-T28G triple mutant and analysed their DNA glycosylase activities (Figure 6A–C). In this study, we analyzed the effects of mutations using the Y68F mutant as a control; the F68F mutant shows approximately the same sequence-specific dsDNA binding ability as that of the wild-type R.PabI, but the Y68F mutant exhibits a reduced catalytic activity (11). Because Y68 is located away from the wedge loop (Figure 4B), the Y68F mutation is predicted not to affect the results of mutation assays of the wedge loop. The results of the DNA glycosylase assay of the Y68F-P27G mutant showed that the activity of the Y68F-P27G mutant decreased to 31% that of the control Y68F mutant. The enzymatic activity of R.PabI was further decreased by the Y68F-P27G-T28G mutation; the DNA glycosylase activity of Y68F-P27G-T28G was not detected at 50°C. These results are consistent with the structural observation that the characteristic structure of the wedge loop is important for the R.PabI activity.

The R.PabI–product–dsDNA complex structure shows that P27 and T28 do not form any direct interactions with the R.PabI recognition sequence (11). To analyse the importance of the wedge loop for the sequence-specific dsDNA binding ability of R.PabI, we performed EMSA using the Y68F and Y68F-P27G-T28G mutants of R.PabI. The EMSA results showed that the sequence-specific dsDNA binding ability of R.PabI(Y68F) was highly reduced by the P27G-T28G mutation (Figure 6D, E). DNA bending is indispensable for the sequence-specific dsDNA binding by R.PabI (Figure 1B). The P27G-T28G mutation is predicted to destabilize the bent dsDNA structure. We also performed a DNA glycosylase activity assay and EMSA using the K154A and K154A-P27G-T28G mutants of R.PabI. The K154A mutant of R.PabI has been used for the structure determination of the R.PabI–product–dsDNA complex. However, the K154A mutant exhibits reduced sequence-specific dsDNA binding ability because K154 is used to stabilize the highly bent and unwound dsDNA structure (Figure 1B) (11). Similar to the results of the Y68F-P27G-T28G mutant, the DNA glycosylase activity of R.PabI(K154A) was reduced by the P27G-T28G mutation (Figure 6C). The results of EMSA using the sequence-specific probe and the nonspecific probe (Supplementary Figure S1B, C) showed that the fractions of the R.PabI-bound dsDNA were reduced by the P27G-T28G mutation (Figure 6F, G). This suggests that the P27G-T28G mutation negatively affects the sequence-specific and/or nonspecific dsDNA binding ability of R.PabI. In contrast to the results of EMSA using R.PabI(Y68F), two shifted bands were observed; the shifted band and the super-shifted band are predicted to be dsDNA–one R.PabI dimer complex and dsDNA–two R.PabI dimer complex, respectively. Because the K154A mutation reduces the sequence-specific dsDNA binding ability of R.PabI, it is unclear whether each shifted band corresponds to the nonspecific dsDNA binding state, the intermediate states or the sequence-specific dsDNA binding state. However, the reduced dsDNA binding ability of the K154A-P27G-T28G mutant suggests that the P27G-T28G mutation destabilizes the bent dsDNA structure because T28 is only used for dsDNA binding in the intermediate state and the product dsDNA binding state (Supplementary Figure S5).

## DISCUSSION

**Figure 7.**
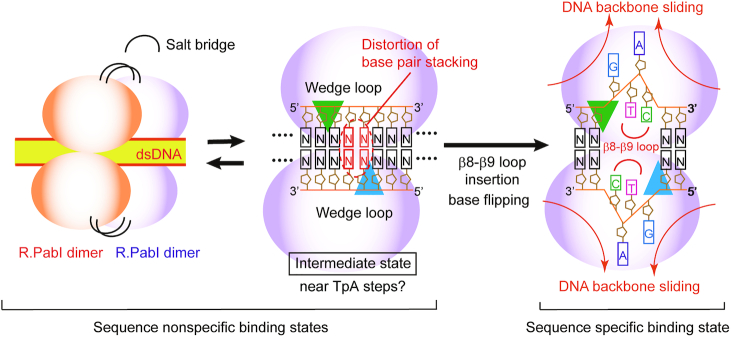
Plausible DNA-binding mechanism of R.PabI. On nonspecific dsDNA, R.PabI binds to dsDNA as a homotetramer or a homodimer. In the intermediate state, R.PabI inserts the wedge loops into dsDNA to stabilize the largely expanded minor groove. When the 5′-GTAC-3′ sequence is located near the largely expanded minor groove region, the β8-β9 loop of R.PabI is predicted to be inserted into the minor groove to recognize and cleave the specific sequence.

In this study, we determined the crystal structures of the R.PabI(Y68F-K154A) mutant in complex with dsDNA. In the course of the experiments, we used dsDNA sequences that possess the R.PabI recognition sites for cocrystallization because we tried to determine the structure of the substrate dsDNA-binding state of R.PabI (not the product dsDNA-binding state of R.PabI that was determined in our previous study (11)). However, the structures of the R.PabI(Y68F-K154A)-dsDNA(GTAC-3 bp-GTAC) and R.PabI(Y68F-K154A)-dsDNA(GTAC-5 bp-GTAC) complexes show that R.PabI does not form any interactions with the R.PabI recognition sequence even if the sequence is located near the HALFPIPE region of the bound R.PabI dimer. Although the data quality was low, we also obtained the R.PabI(Y68F-K154A)–dsDNA(nonspecific) complex structure, which has approximately the same structure as the dsDNA(GTAC-3 bp-GTAC) and dsDNA(GTAC-3 bp-GTAC) complexes (Supplementary Figure S2A). These results indicate that the structures of the R.PabI(Y68F-K154A)–dsDNA complexes determined in this study are one form of the sequence-nonspecific dsDNA-binding states of R.PabI (the intermediate state). Sequence-nonspecific dsDNA binding states of DNA binding proteins are important to facilitate diffusion on DNA. For example, EcoRV and BamHI, which belong to the PD-(D/K)XK superfamily of restriction enzymes, weakly bind to the nonspecific dsDNA sequence and diffuse along dsDNA to search their recognition sequence (4,5,36). In our previous study, we also determined the crystal structure of the other nonspecific dsDNA-binding state of R.PabI in which two R.PabI dimers form a tetrameric structure to sandwich dsDNA. The tetrameric structure of R.PabI on sequence-nonspecific dsDNA is important to facilitate diffusion on dsDNA (25). R.PabI bends and unwinds dsDNA at the recognition sequence by the insertion of the β8–β9 loop from the minor groove side of dsDNA (11). In the intermediate state, the bound dsDNA is drastically bent by the binding of R.PabI and the base stacking in the bent dsDNA is distorted (Figures 2 and 5). R.PabI is predicted to utilize two sequence-nonspecific dsDNA-binding states for its activity (Figure 7).

R.PabI drives wedge loops into the minor groove of dsDNA and stabilizes the largely expanded minor groove structure (Figure 4). The wedge loop residues are important for the DNA glycosylase activity and the sequence-specific dsDNA binding ability of R.PabI (Figure 6). In the intermediate state, there are three base pairs between the two wedge-loop inserted regions. In contrast, in the R.PabI-product dsDNA complex, there are four base pairs of the R.PabI recognition sequence between the two wedge-loop inserted regions (Figure 7 and Supplementary Figure S6). When the β8-β9 loop is inserted into the recognition sequence, the DNA backbone bound in the HALFPIPE region is predicted to slide on the electropositive protein surface to pull out adenine and guanine bases into their recognition sites on the R.PabI surface. DNA repair enzymes have been predicted to detect their targets by searching for DNA flexibility caused by weakened base stackings of the lesion sites (37–40). Among all dinucleotide steps in dsDNA, the TpA (5′-Thy-Ade-3′) step shows the lowest stacking energy (41). The TpA step in the R.PabI recognition sequence (5′-GTAC-3′) will be highly distorted when the TpA step is located at the largely expanded minor groove region that is stabilized by the wedge loops. In fact, the roll angle at the base-stacking distorted region is larger in the dsDNA(GTAC-3 bp-GTAC) complex in which the TpA step (Thy-4 to Ade-3) exists at the distorted region (Figure 5). R.PabI tightly recognizes the flipped guanine base of the recognition sequence using the guanine recognition site on the protein surface (Figure 1A) (11). R.PabI is predicted to detect the flexibility of the TpA step in the drastically bent dsDNA and to recognize the specific sequence using the β8–β9 loop when Gua and Cyt exist upstream and downstream of the TpA step, respectively; the formation of the intermediate state is predicted to be important for the indirect readout of the R.PabI recognition sequence on which further selection steps (flipping out of bases) are built. A similar indirect readout mechanism is also observed in the type II restriction endonuclease HincII (42). In contrast to DNA repair enzymes that recognize DNA lesions, R.PabI recognizes normal DNA bases in a sequence-dependent manner. The DNA bending by R.PabI is predicted to be important to emphasize the DNA flexibility of the TpA step. In general, TpA steps in dsDNA tend to widen the minor groove of dsDNA due to its poor stacking energy (43). In the intermediate state, R.PabI binds to the highly expanded minor groove structure of dsDNA. The tetrameric structure of R.PabI on nonspecific dsDNA may be dissociated into the intermediate state at TpA steps in dsDNA (Figure 7).

Certainly, we cannot exclude the possibility that the intermediate structure observed in this study is an artefact of mutagenesis and/or crystal packing. However, for at least three reasons, it is reasonable to conclude that the intermediate state is the ‘on-pathway’ structure during DNA bending by R.PabI. First, the intermediate state exhibits both characteristics of the sequence-nonspecific dsDNA binding state and the product dsDNA binding state; the intermediate state does not form any sequence-specific interactions with dsDNA like the nonspecific dsDNA binding state; the bound dsDNA structure in the intermediate state shows high similarity to that in the product dsDNA binding state (Figure 2B). Second, the distortion of the base stacking observed in the intermediate state seems to be energetically favourable to unwind the R.PabI recognition sequence and flip out DNA bases. Finally, the importance of the wedge loop was confirmed by EMSA and DNA glycosylase activity assays using both the Y68F and K154A mutants of R.PabI (Figure 6). This suggests that the importance of the wedge loop is not affected by the mutations. R.PabI is predicted to form the sequence-specific interaction with dsDNA through the intermediate structure (Figure 7).

In this study, we have shown the distortion of dsDNA structures by the binding of the R.PabI(Y68F-K154A) mutant. HALFPIPE superfamily enzymes are also conserved in *Campylobacter coli* and *Helicobacter pylori* (Supplementary Figure S5). These homologs also recognize the sequence 5′-GTAC-3′ like R.PabI (44). However, residues that are utilized for dsDNA recognition, including the residues of the wedge loop, are poorly conserved among the homologues, although the catalytic residues are highly conserved. This may suggest that the R.PabI homologues from *C. coli* and *H. pylori* adopt different DNA recognition mechanisms. The DNA recognition mechanisms of these homologous proteins will be clarified by determining their structures.

## DATA AVAILABILITY

Atomic coordinates and structure factors for the reported crystal structures have been deposited with the Protein Data Bank under accession numbers 6L2N (the dsDNA(GTAC-3 bp-GTAC) complex), 6L2O (the dsDNA(GTAC-5 bp-GTAC) complex) and 6M3L (the dsDNA(nonspecific) complex).

## Supplementary Material

gkaa184_Supplemental_File
