# Supplementary material for: Distortion of double-stranded DNA structure by the binding of the restriction DNA glycosylase R.PabI
Source: Nucleic Acids Res. 2020 Mar 31;48(9):5106–18. doi: 10.1093/nar/gkaa184 (PMC7229829; doi:10.1093/nar/gkaa184)
Supplement: gkaa184_Supplemental_File [file gkaa184_supplemental_file.pdf]

Supplementary Materials

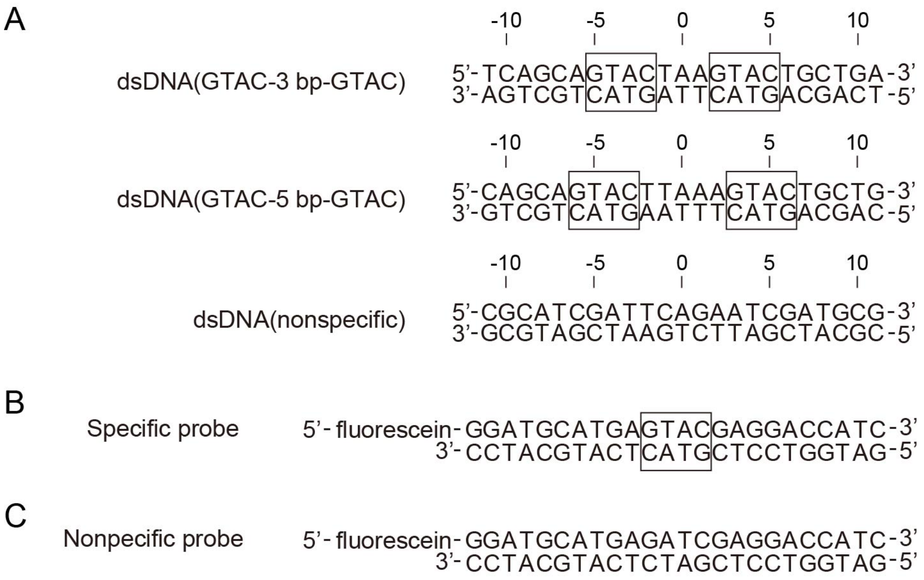

Supplementary Figure 1. dsDNA sequences used in this study. (A) dsDNA sequences (23 bp) used for cocrystallization assays. The R.PabI recognition sequence (5'-GTAC-3') is indicated by boxes. (B) A dsDNA sequence (24 bp) used for the DNA glycosylase activity assay and the EMSA (the specific probe). (C) A dsDNA sequence (24 bp) used for the EMSA (the nonspecific probe).

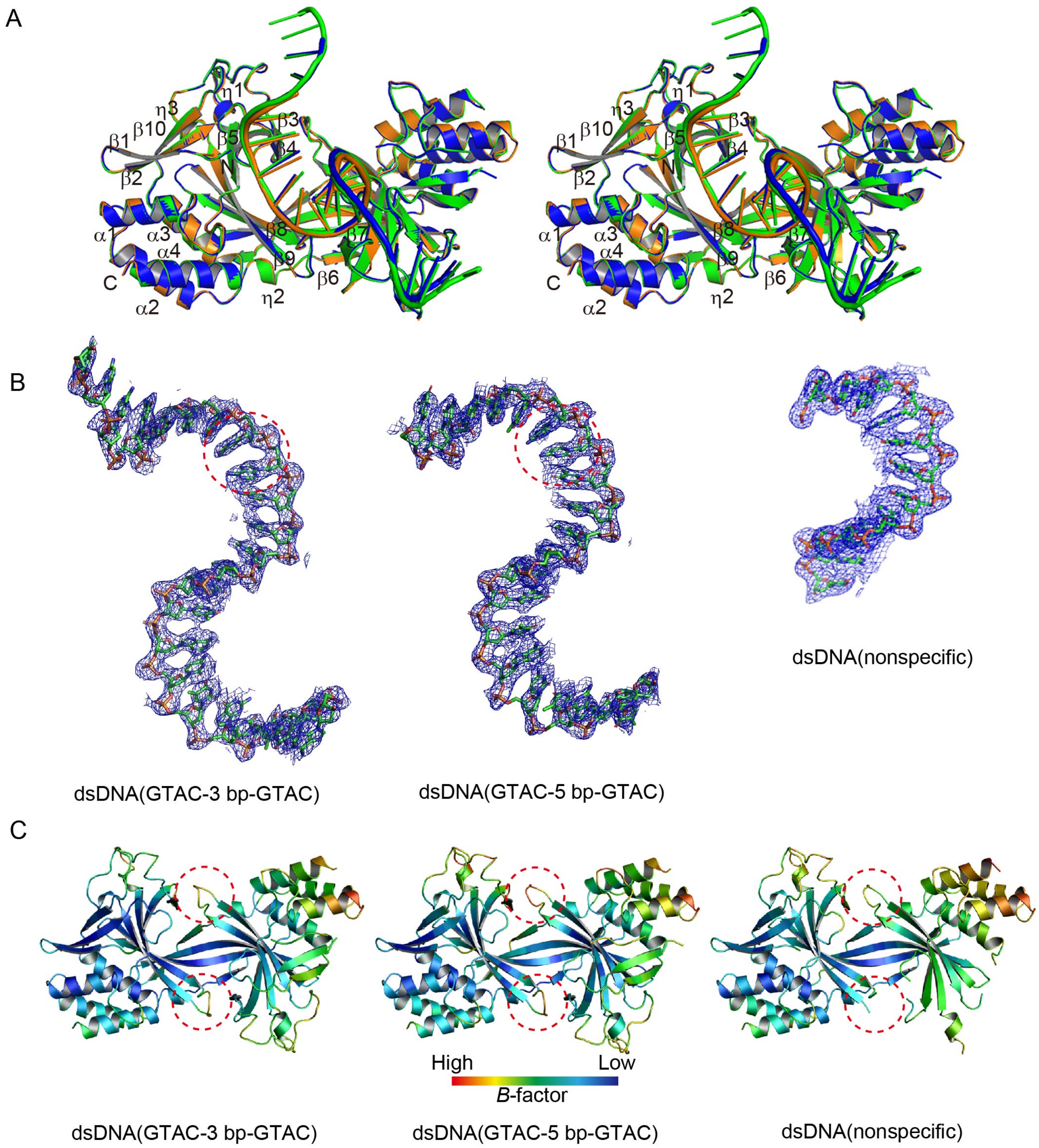

Supplementary Figure 2. Structure determination of the R.PabI(Y68F-K154A)-dsDNA complexes. (A) The stereo diagram of the superposition of the R.PabI(Y68F-K154A)-dsDNA complexes in the asymmetric units; green, the dsDNA(GTAC-3 bp-GTAC) complex; blue, the dsDNA(GTAC-5 bp-GTAC) complex; orange, the dsDNA(nonspecific) complex. Secondary structure assignments are labelled on the model. (B) The structure of DNA in each complex is shown in stick model. The composite omit map (sigma-A weighted $2F_O$-F_C, 1 σ) is shown in blue mesh. The positions at which base-pair stacking is distorted are indicated by red dotted circles. (C) Temperature factors of R.PabI dimers in the R.PabI(Y68F-K154A)-dsDNA complexes. The positions of the β8-β9 loop are indicated by red doted circles.

A

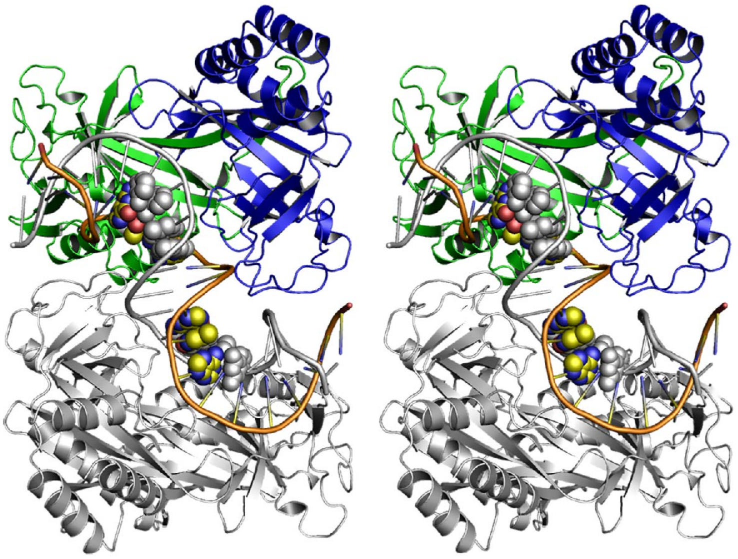

dsDNA(GTAC-3 bp-GTAC)

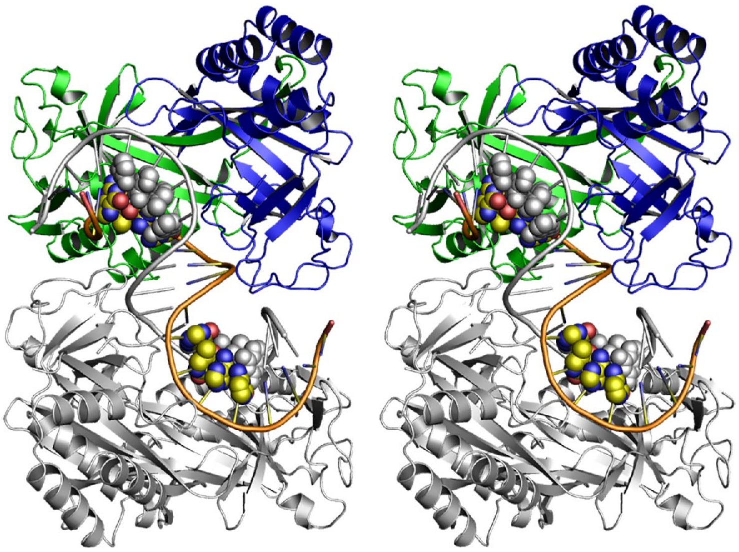

dsDNA(GTAC-5 bp-GTAC)

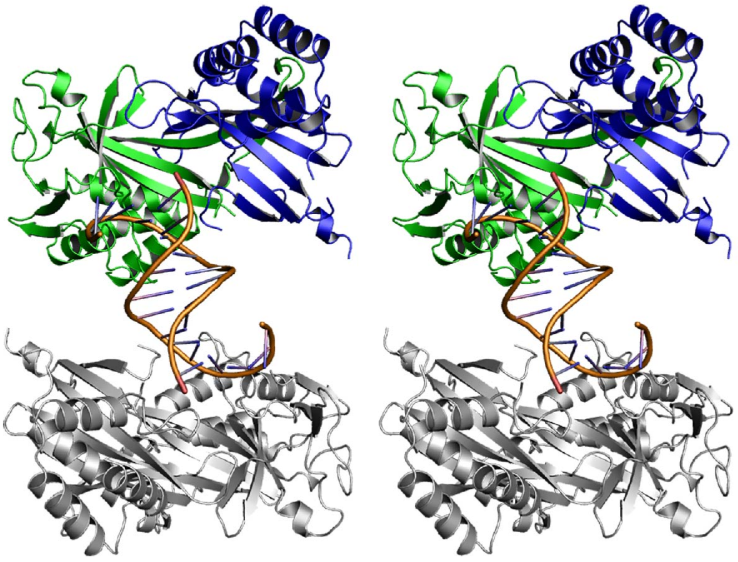

dsDNA(nonspecific)

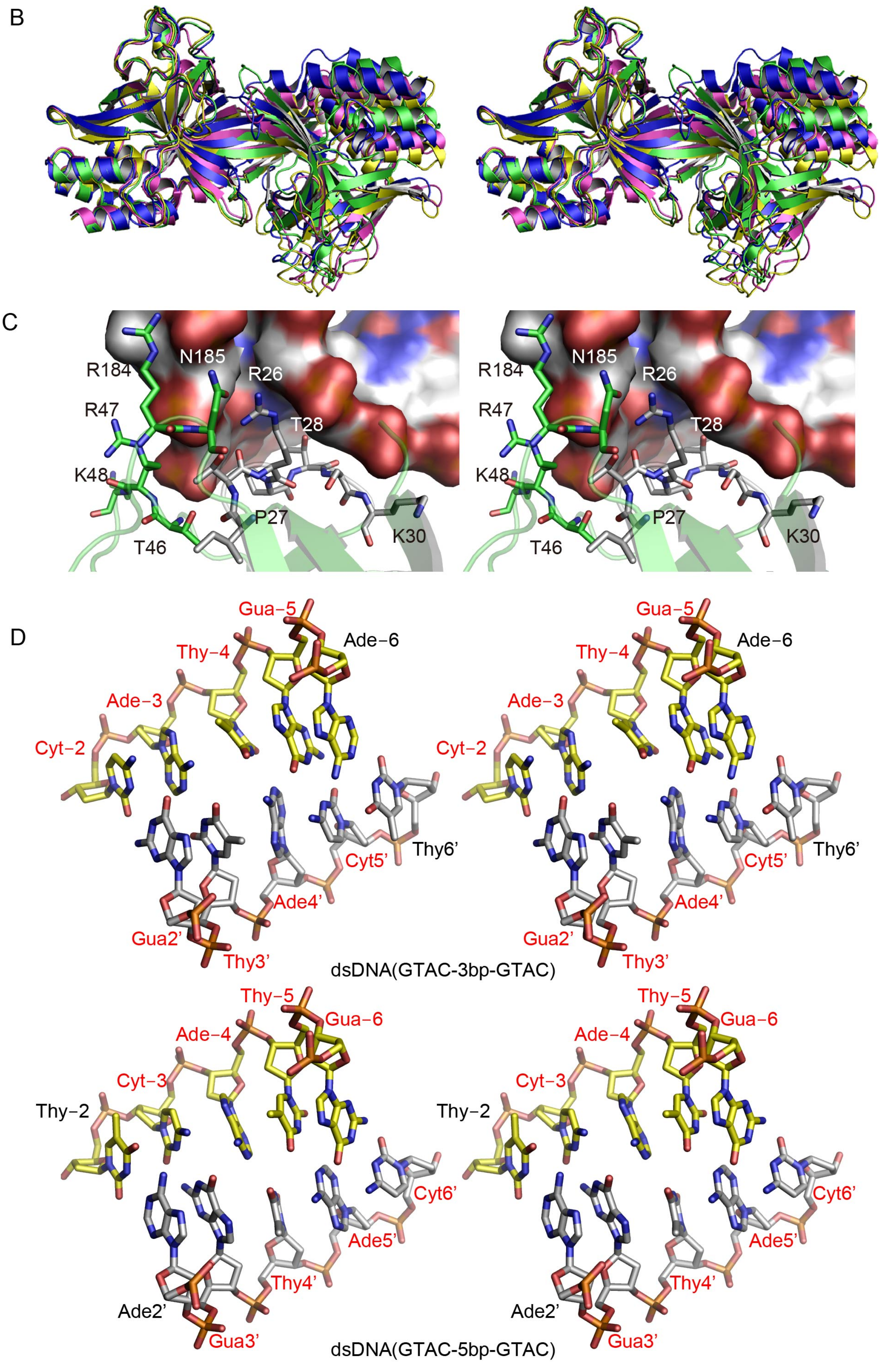

Supplementary Figure 3. Stereo diagrams. (A) Stereo diagrams of Figure 2A. (B) Stereo diagram of Figure 3A. (C) Stereo diagram of Figure 4D. (D) Stereo diagrams of Figure 5A.

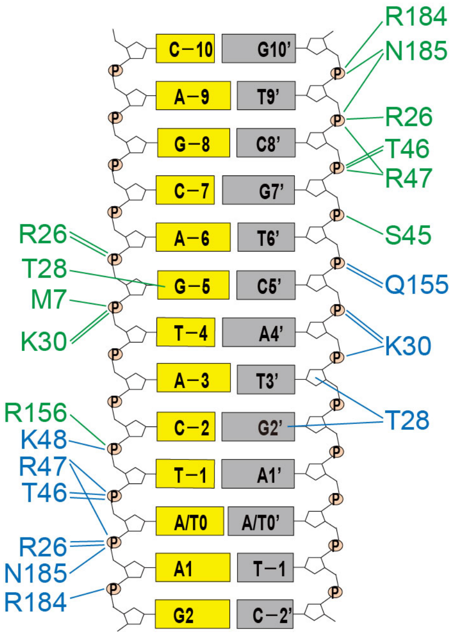

Supplementary Figure 4. dsDNA(GTAC-3 bp-GTAC) recognition by the R.PabI(Y68F-K154A) mutant. Residues of chains A and B are shown in green and blue texts, respectively. Intermolecular hydrogen bonds between R.PabI and dsDNA are shown in green (R.PabI chain A-dsDNA) and blue (R.PabI chain B-dsDNA) lines.

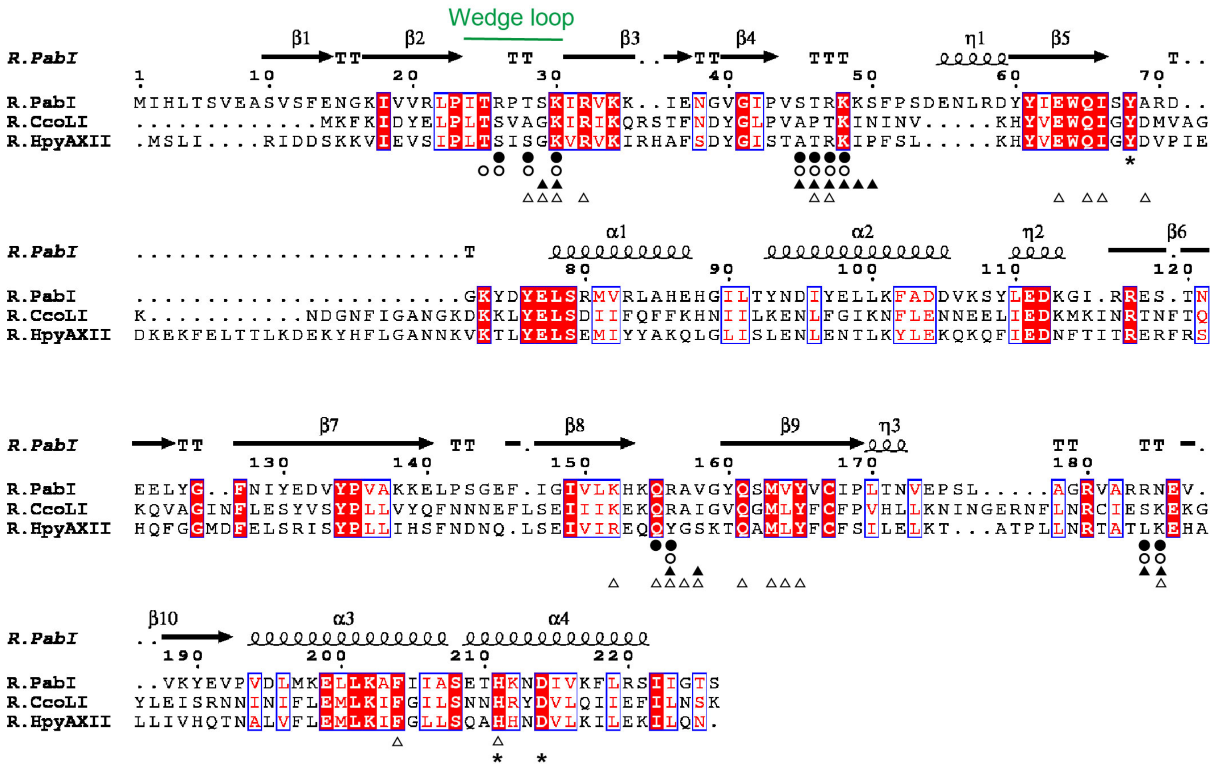

Supplementary Figure 5. Amino acid sequence alignments of R.PabI homologues (R.CcoLI from *Campylobacter coli*, WP_002830209; R.HpyAXII from *Helicobacter pylori*, ACI43084.1). Invariant residues are highlighted with red boxes, and conserved residues are shown in red text. Residues that form hydrogen bonds with DNA in the dsDNA(GTAC-3 bp-GTAC) and dsDNA(GTAC-5 bp-GTAC) complexes are marked with black and open circles, respectively. Residues that form hydrogen bonds in the sequence-nonspecific dsDNA-binding state (PDB ID: 5IFF) and the product dsDNA-binding state (PDB ID: 3WAZ) are marked with black and open triangles, respectively. The catalytic resides are marked with asterisks. The secondary structure of R.PabI in the dsDNA(GTAC-5 bp-GTAC) complex is indicated by helices (α and 3_{10} (η) helices), arrows (β strands) and TT (β-turn).

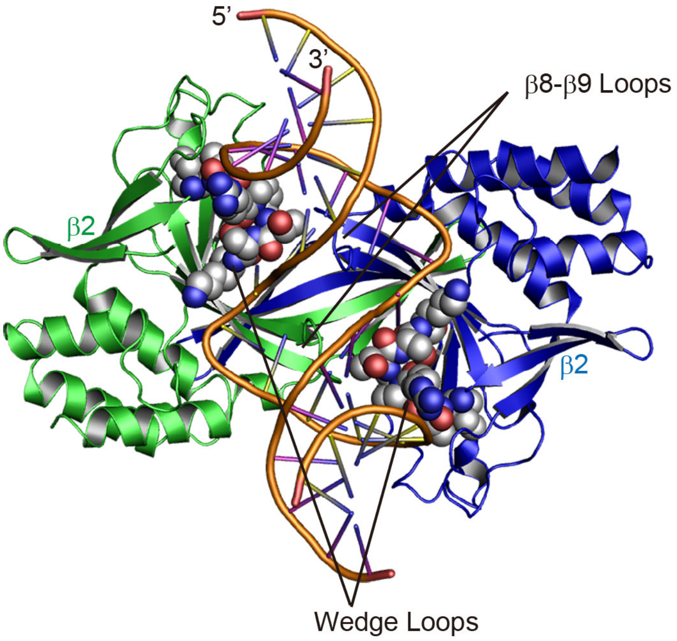

Supplementary Figure 6. Wedge loops in the R.PabI-product dsDNA complex (PDB ID: 3WAZ). The R.PabI dimer is coloured green (chain A) and blue (chain B). The bound dsDNA is coloured orange. Residues in the wedge loop are shown in sphere models. The positions of the β8-β9 loop, which is utilized for base flipping, are also indicated.

Table S1. Hydrogen bonds between R.PabI(Y68F-K154A) and dsDNA(GTAC-5 bp-GTAC)

Protein				H-bonds	DNA			
Chain ID	Residue	Main/ Side*	Atom Name	Distance (Å)	Chain ID	Base	Base/ Backbone	Atom Name
A	M7	Main	N	2.63	C	Ade−4	Backbone	OP1
A	R26	Side	NE	3.10	C	Gua−6	Backbone	O3’
A	R26	Side	NE	3.28	C	Thy−5	Backbone	OP2
A	T28	Side	OG1	2.72	C	Thy−5	Base	O2
A	K30	Main	N	3.35	C	Ade−4	Backbone	OP2
B	R26	Side	NH1	3.33	C	Ade0	Backbone	O3’
B	R26	Side	NH1	3.26	C	Ade1	Backbone	OP2
B	T46	Main	N	2.78	C	Ade0	Backbone	OP2
B	T46	Side	OG1	3.31	C	Ade0	Backbone	OP2
B	R47	Main	N	3.35	C	Ade0	Backbone	OP2
B	R184	Side	NH1	3.64	C	Ade2	Backbone	OP2
B	N185	Main	N	2.70	C	Ade1	Backbone	OP2
A	T25	Side	OG1	2.81	C’	Cyt9’	Backbone	OP1
A	R26	Side	NH1	3.75	C’	Gua8’	Backbone	O3’
A	R26	Side	NH1	3.44	C’	Cyt9’	Backbone	OP1
A	S45	Side	OG	3.58	C’	Thy7’	Backbone	O5’
A	T46	Main	N	3.12	C’	Gua8’	Backbone	OP1
A	T46	Side	OG1	3.58	C’	Gua8’	Backbone	OP1
A	R47	Main	N	3.63	C’	Gua8’	Backbone	OP1
A	R47	Main	N	3.49	C’	Gua8’	Backbone	OP2
A	R47	Side	NE	3.06	C’	Cyt9’	Backbone	OP2
A	K48	Main	N	3.72	C’	Gua8’	Backbone	OP2
A	R184	Side	NE	2.71	C’	Thy10’	Backbone	OP2
A	R184	Side	NH2	2.53	C’	Thy10’	Backbone	OP2
A	N185	Main	N	2.85	C’	Cyt9’	Backbone	OP1
A	N185	Side	ND2	3.21	C’	Thy10’	Backbone	OP2
B	M7	Main	N	2.94	C’	Thy4’	Backbone	OP2
B	M7	Main	N	3.83	C’	Gua3’	Backbone	O5’
B	T28	Side	OG1	3.04	C’	Gua3’	Backbone	O4’
B	K30	Main	N	3.53	C’	Thy4’	Backbone	OP1
B	R156	Side	NE	3.67	C’	Cyt6’	Backbone	O3’

DNA bases of the symmetrically related molecule are indicated by a prime.
*Main, main chain; Side, side chain.

Table S2. Hydrogen bonds between R.PabI(Y68F-K154A) and dsDNA(GTAC-3 bp-GTAC)

Protein				H-bonds	DNA			
Chain ID	Residue	Main/ Side*	Atom Name	Distance (Å)	Chain ID	Base	Base/ Backbone	Atom Name
A	M7	Main	N	2.88	C	Thy−4	Backbone	OP1
A	R26	Side	NE	3.24	C	Ade−6	Backbone	O3’
A	R26	Side	NE	3.34	C	Gua−5	Backbone	OP2
A	T28	Side	OG1	3.29	C	Gua−5	Base	N2
A	K30	Main	N	3.31	C	Thy−4	Backbone	OP2
A	K30	Side	NZ	2.84	C	Thy−4	Backbone	OP2
A	R156	Side	NE	3.32	C	Cyt−2	Backbone	O3’
B	R26	Side	NH2	3.29	C	Ade0	Backbone	O3’
B	R26	Side	NH2	2.82	C	Ade1	Backbone	OP2
B	T46	Main	N	2.93	C	Ade0	Backbone	OP2
B	T46	Side	OG1	3.52	C	Ade0	Backbone	OP2
B	R47	Main	N	3.75	C	Ade0	Backbone	OP2
B	R47	Side	NE	3.49	C	Ade1	Backbone	OP1
B	K48	Side	NZ	3.85	C	Thy−1	Backbone	O5’
B	R184	Side	NH1	2.27	C	Gua2	Backbone	OP2
B	N185	Main	N	2.91	C	Ade1	Backbone	OP2
A	R26	Side	NH1	3.30	C’	Thy9’	Backbone	OP1
A	S45	Side	OG	3.89	C’	Gua7’	Backbone	O5’
A	T46	Main	N	3.39	C’	Cyt8’	Backbone	OP1
A	T46	Side	OG1	3.77	C’	Cyt8’	Backbone	OP1
A	R47	Main	N	3.45	C’	Cyt8’	Backbone	OP1
A	R47	Side	NE	2.93	C’	Thy9’	Backbone	OP2
A	R184	Side	NH1	3.09	C’	Gua10’	Backbone	OP2
A	N185	Main	N	3.28	C’	Thy9’	Backbone	OP1
A	N185	Side	ND2	3.25	C’	Gua10’	Backbone	OP2
B	T28	Side	OG1	3.82	C’	Gua2’	Base	N2
B	T28	Side	OG1	2.85	C’	Thy3’	Backbone	O4’
B	K30	Main	N	3.81	C’	Ade4’	Backbone	OP1
B	K30	Side	NZ	2.72	C’	Cyt5’	Backbone	OP1
B	K30	Side	NZ	3.22	C’	Ade4’	Backbone	O3’
B	Q155	Side	NE2	2.66	C’	Thy6’	Backbone	OP1
B	Q155	Side	NE2	3.58	C’	Cyt5’	Backbone	O3’

DNA bases of the symmetrically related molecule are indicated by a prime.
*Main, main chain; Side, side chain.
